# American Dental Association

**Published:** 1866-09

**Authors:** 


### Editorial.

### AMERICAN DENTAL ASSOCIATION.

THE annual meeting of this body was held in Boston, beginning on Tuesday, the —th of July, and continued in session seven days; three days longer than ever before.

A large amount of business was brought before the meeting, and a great deal was accomplished, notwithstanding some complained that more was not done; but it should be remembered that large bodies do not transact business with the rapidity of smaller ones.

The American Medical Association became apprised of that fact some time ago, and introduced such arrangements as to obviate the difficulty. And it may become necessary in the future for the American Dental Association to do more of its business through committees, or sections; of this, however, the necessities of the future will indicate.

Great harmony and unanimity prevailed in the accomplishment of all the strictly legitimate work of the Association.

It would be impossible here to speak of, much less to present in detail, all that was done. Quite a number of strictly professional subjects were presented and discussed. There were some, however, that could hardly be regarded as professional, that came in for their full share of consideration.

The rubber question ("*Monsieur Tonson come again*") came up in all its ruggedness, and demanded and received of the Association much consideration and time.

This is one of the subjects that refuse to be settled. It yet remains to be seen whether the action then had will prove a finality. But of this subject we will have more to say in the future.

Another very important item of business accomplished was the preparation and adoption of a *Code of Ethics.* This, we think, will exert a great influence for good upon the profession. It fixes a definite standard of professional conduct, and will be a very happy guide for all those engaged in associated efforts for the elevation and advancement of the profession.

This code should, and doubtless will, be adopted by all local societies, and especially by those represented in the National Association, for it would be altogether inconsistent for any society that would not recognize and be governed by such code to be represented in the central body.

The American Dental Association is made up of delegates from the local societies, and these delegates are the persons who adopted this code, and they are necessarily bound by it; but it would be wholly inconsistent for such persons only as had been delegates to be subject to the wholesome regulations of this code, while their fellow members of their respective local societies who had not been delegates regarded themselves as not amenable to it or bound by it, but could with impunity run counter to its wholesome rules. We think this view must be taken of it by all local societies, and that all will adopt it. We do not think the Central Association could or would admit delegates hereafter from a society that would not adopt its code of ethics.

Many good papers were read upon the stated subjects by the respective committees, and also quite a number of volunteer papers, many of which were very good.

So much of a purely business character crowded upon the meeting that but little time was afforded for clinic exercises, though ample preparations were made for them. There were on exhibition a great number of instruments and appliances, many of them new, and most of them very good. A good arrangement was made for the exhibition of all these. We think, however, that an improvement might be made in reference to these things, in the following manner: Let there be a committee of three appointed, whose business it shall be to receive all such instruments and appliances, with a full and complete description of each; then, at a time appointed for the purpose, it should be the duty of this committee to exhibit, describe and give all needed information of each article before the Association. Such committee, of course, should have some discretionary power. In this way everything of value would be brought fully to the notice of the Association.

We know that at this meeting—and it has been so before—there were many valuable instruments that were not examined by or described to a large number of the members, which would not be the case under such an arrangement as we suggest.

For the kind regard, attention and hospitality of our brethren of New England, and especially those of Boston and vicinity, we have the highest appreciation and kindest remembrance, and in this we but reiterate the sentiments of all those from a distance. Indeed, it was in every one's *mouth*, as we can testify.

Upon the seventh day of the meeting, in the afternoon, it adjourned to meet in Cincinnati on the last Tuesday of July, 1867. Selecting this place we think one of the best things that was done. Every year the profession look forward to these meetings with an increasing interest.

Quite a number of societies were represented at this meeting which have been organized within the last year; and we hope to see the good work go on with equal if not greater energy for the coming year.

